# Brain Injury-Induced Synaptic Reorganization in Hilar Inhibitory Neurons Is Differentially Suppressed by Rapamycin

**DOI:** 10.1523/ENEURO.0134-17.2017

**Published:** 2017-10-04

**Authors:** Corwin R. Butler, Jeffery A. Boychuk, Bret N. Smith

**Affiliations:** 1 Department of Physiology College of Medicine, University of Kentucky, Lexington, KY 40536; 2 Epilepsy Center, University of Kentucky, Lexington, KY 40536; 3 Spinal Cord and Brain Injury Research Center (SCoBIRC), University of Kentucky, Lexington, KY 40536

**Keywords:** dentate gyrus, epilepsy, interneuron, mossy fiber, mTOR, photostimulation

## Abstract

Following traumatic brain injury (TBI), treatment with rapamycin suppresses mammalian (mechanistic) target of rapamycin (mTOR) activity and specific components of hippocampal synaptic reorganization associated with altered cortical excitability and seizure susceptibility. Reemergence of seizures after cessation of rapamycin treatment suggests, however, an incomplete suppression of epileptogenesis. Hilar inhibitory interneurons regulate dentate granule cell (DGC) activity, and *de novo* synaptic input from both DGCs and CA3 pyramidal cells after TBI increases their excitability but effects of rapamycin treatment on the injury-induced plasticity of interneurons is only partially described. Using transgenic mice in which enhanced green fluorescent protein (eGFP) is expressed in the somatostatinergic subset of hilar inhibitory interneurons, we tested the effect of daily systemic rapamycin treatment (3 mg/kg) on the excitability of hilar inhibitory interneurons after controlled cortical impact (CCI)-induced focal brain injury. Rapamycin treatment reduced, but did not normalize, the injury-induced increase in excitability of surviving eGFP+ hilar interneurons. The injury-induced increase in response to selective glutamate photostimulation of DGCs was reduced to normal levels after mTOR inhibition, but the postinjury increase in synaptic excitation arising from CA3 pyramidal cell activity was unaffected by rapamycin treatment. The incomplete suppression of synaptic reorganization in inhibitory circuits after brain injury could contribute to hippocampal hyperexcitability and the eventual reemergence of the epileptogenic process upon cessation of mTOR inhibition. Further, the cell-selective effect of mTOR inhibition on synaptic reorganization after CCI suggests possible mechanisms by which rapamycin treatment modifies epileptogenesis in some models but not others.

## Significance Statement

Rapamycin inhibits mammalian (mechanistic) target of rapamycin (mTOR) signaling to suppress abnormal patterns of cell growth and circuit reorganization found in pathologies such as acquired epilepsy, tuberous sclerosis, and cancer. Here, rapamycin was studied for its effects on hippocampal circuit reorganization in a model of focal brain injury that leads to spontaneous seizures in a subset of mice. Rapamycin treatment in these animals reduced the injury-induced excitatory synaptic input from DGCs onto surviving hilar interneurons, but failed to prevent the backprojection of CA3 pyramidal cells onto these interneurons seen after brain injury. These findings indicate that mTOR inhibition fails to ubiquitously suppress recurrent excitatory synaptic reorganization after brain injury, and suggests that rapamycin’s effects on reactive axonal plasticity in the hippocampal formation are cell-type specific.

## Introduction

Functional reactive plasticity of dentate granule cells (DGCs), CA3 pyramidal cells, and hilar inhibitory neurons has been associated with epilepsy development after severe traumatic brain injury (TBI; Scharfman, 2007; Hunt et al., 2009; Halabisky et al., 2010; Hunt et al., 2010, 2011; Pavlov et al., 2011; Gupta et al., 2012; Guo et al., 2013). Although the nature of their contribution is controversial, the sprouting of DGC axons (i.e., mossy fiber sprouting; MFS) may contribute to the formation of an epileptogenic circuit. Increased excitability of somatostatinergic hilar inhibitory interneurons is also a component of this synaptic reorganization (Halabisky et al., 2010; Hunt et al., 2011). These interneurons function to inhibit DGC activity after perforant pathway activation, and are particularly susceptible to cell death after TBI (Lowenstein et al., 1992; Hicks et al., 1993; Butler et al., 2016). After an epileptogenic insult, increased synaptic input arising *de novo* from both DGCs and CA3 pyramidal neurons drives the increased excitability of surviving hilar interneurons (Halabisky et al., 2010; Hunt et al., 2011). The reorganization of excitatory synaptic input to hilar inhibitory interneurons is therefore a component of the altered excitation-inhibition balance in the hippocampus associated with epileptogenesis after TBI, and hyperexcitability of inhibitory neurons may promote epileptiform activity (Yekhlef et al., 2015; Shiri et al., 2017).

Treatment with the mammalian (mechanistic) target of rapamycin (mTOR) inhibitor, rapamycin in the controlled cortical impact (CCI) model of posttraumatic epilepsy (PTE) reduces spontaneous seizure development (Guo et al., 2013; Butler et al., 2015). Rapamycin also suppresses several cellular correlates of epileptogenesis, including axon remodeling in the dentate gyrus after CCI and in the pilocarpine-induced status epilepticus (SE) model of temporal lobe epilepsy (TLE; Buckmaster et al., 2009; Buckmaster and Lew, 2011; Buckmaster and Wen, 2011; Guo et al., 2013; Butler et al., 2015; Yamawaki et al., 2015), but other effects of mTOR inhibition on TBI-induced cortical synaptic plasticity are not well understood. Accordingly, cessation of treatment results in the reemergence of seizures and synaptic reorganization (Buckmaster et al., 2009; Guo et al., 2013), and epileptogenesis is not prevented in some models (Heng et al., 2013), suggesting rapamycin treatment may alleviate only a subset of the functional cellular changes underlying epileptogenesis. For example GABA_A_ receptors undergo functional changes after CCI that persist for months after the injury (Boychuk et al., 2016) and these changes are not universally constrained by rapamycin treatment (Butler et al., 2016). Additionally, the *de novo* emergence of convergent synaptic inputs onto surviving inhibitory neurons after TBI could powerfully affect hippocampal function (Hunt et al., 2011), but mTOR’s involvement in this synaptic remodeling is unknown. Here, we used transgenic mice in which somatostatinergic hilar inhibitory interneurons express enhanced green fluorescent protein (eGFP) (Oliva et al., 2000) to study effects of mTOR inhibition on reorganization of excitatory synaptic input to hilar inhibitory interneurons after CCI injury. We tested the hypothesis that continual rapamycin treatment after CCI obviates injury-induced formation of new excitatory synaptic connections arising from both DGCs and CA3 pyramidal cells onto surviving hilar inhibitory interneurons.

## Materials and Methods

### Animals

Male FVB-Tg(GadGFP)4570Swn/J mice (i.e., GIN mice; The Jackson Laboratory) age six to eight weeks old, weighing 23–28 g, or male CD-1 mice (Harlan) age six to eight weeks old, weighing 30–35 g, were housed in a normal 14/10 h light/dark cycle. Mice were housed in the University of Kentucky vivarium for a minimum of 7 d before experimentation; food and water was provided *ad libitum*. GIN mice express eGFP in the somatostatinergic subset of GABAergic interneurons in cortical brain regions; in the hilus, somatostatin is expressed in ∼95% of eGFP-labeled (eGFP+) hilar interneurons (Oliva et al., 2000). All procedures were approved by the University of Kentucky Animal Care and Use Committee and followed the National Institutes of Health guidelines for the care and treatment of animals.

### TBI

Mice were subjected to severe unilateral, cortical contusion injury by CCI, as described previously (Dixon et al., 1991; Scheff et al., 1997; Hall et al., 2005; Hunt et al., 2009; Guo et al., 2013; Butler et al., 2015). Briefly, mice were anesthetized by 2% isoflurane inhalation and placed in a stereotaxic frame. The skull was exposed by midline incision, and ∼5 mm craniotomy was made lateral to the sagittal suture, centered between bregma and lambda. The skull cap was carefully removed, avoiding damage to the exposed underlying dura. The contusion device consisted of a computer-controlled, pneumatically driven impactor fitted with a beveled stainless-steel tip 3 mm in diameter (TBI-0310, Precision Systems and Instrumentation). A focal, nonpenetrating brain injury was delivered to compress the cortex 1.0 mm in depth (hard stop) at a velocity of 3.5 m/s and 500 ms duration. A qualitative postoperative health assessment was performed daily for 4 d after CCI and periodically thereafter.

### Rapamycin treatment

Rapamycin (LC Labs) was initially dissolved in 100% ethanol (20 mg/ml), stored at −20°C, and diluted in a vehicle solution containing 5% Tween 80, 5% PEG 400, and 4% ethanol in distilled, deionized water immediately before injection (Guo et al., 2013; Heng et al., 2013; Butler et al., 2015). Rapamycin (3 mg/kg; i.p.) or vehicle was injected after mice regained consciousness following CCI injury (20–30 min) and continued once daily until the mouse was used for experimentation. All other chemicals were obtained from Fischer Scientific unless otherwise indicated.

### Slice preparation

Mice were deeply anesthetized by isoflurane inhalation to effect (lack of tail pinch response) and decapitated while anesthetized. The brain was removed and placed in ice-cold (2–4°C) artificial CSF (ACSF) containing 124 mM NaCl, 3 mM KCl, 2 mM CaCl_2_, 26 mM NaHCO_3_, 1.3 mM MgCl_2_, 11 mM glucose, and 1.4 mM NaH_2_PO_4_ equilibrated with 95% O_2_-5% CO_2_ (pH 7.2–7.4). Brains were blocked, glued to a sectioning stage, and 350-μm-thick coronal slices were cut in cold, oxygenated ACSF using a vibrating microtome (Vibratome Series 1000; Technical Products International). The hippocampus was isolated from surrounding tissue, making sure to completely remove the entorhinal cortex, thereby avoiding synaptic excitation of hilar inhibitory interneurons by activity of intact cortical neurons mediating performant path input. In a subset of slices, a scalpel cut was made across the hilus to isolate the dentate gyrus from the CA3 pyramidal cell layer to abrogate action potential-dependent excitatory neurotransmission in GFP+ hilar interneurons that arose from CA3. Slices were transferred to a storage chamber containing oxygenated warmed ACSF (32–34°C). Slices of the septal and temporal hippocampus from the hemispheres ipsilateral and contralateral to CCI injury were used in these experiments and compared to comparable slices from sham-injured mice (i.e., craniotomy, but no impact injury).

### Whole-cell patch-clamp and on-cell recordings

After an equilibration period of at least 1 h, a single slice was transferred to a recording chamber on an upright, fixed-stage microscope equipped with epifluorescence and infrared, differential interference contrast optics (IR-DIC; Olympus BX50WI), where it was continuously superfused with warmed (32–34°C) oxygenated ACSF. Recordings were performed from eGFP-labeled (i.e., eGFP+) hilar interneurons, identified by epiflorescence illumination, and DGCs or CA3 pyramidal cells identified using DIC imaging. Recording pipettes were made from borosilicate glass (1.65 mm outer diameter, 0.45 mm inner diameter; King Precision Glass) with a P-87 horizontal puller (Sutter Instrument). The intracellular solution contained 130 mM K^+^-gluconate, 1 mM NaCl, 5 mM EGTA, 10 mM HEPES, 1 mM MgCl_2_, 1 mM CaCl_2_, 3 mM KOH, and 2 mM ATP. Open tip resistance was 2–5 MΩ. Recordings were obtained using an Axon Axopatch 200B or Axon Multiclamp 700B amplifier (Molecular Devices), low-pass filtered at 2–5 kHz, digitized at 20 kHz with a Digidata 1322A or Digidata 1550A (Molecular Devices), and acquired using pClamp 10.2 or 10.5 programs (Clampfit, Molecular Devices). On-cell recordings of spontaneous sodium currents mediating action potential firing were made with no holding command. In a subset of recordings, the ionotropic glutamate receptor antagonist, kynurenic acid (30 μM) was added to the ACSF. Whole-cell patch-clamp recordings of spontaneous and evoked EPSCs (sEPSCs and eEPSCs) were made in voltage-clamp mode at −70mV. EPSCs were identified as transient inward currents having a rapid rise and single exponential decay. EPSCs were analyzed using MiniAnalysis software (version 6.0.3, Synaptosoft; Decatur GA) and only included events with amplitudes >3× RMS.

### Glutamate photostimulation

Slices were superfused with 4-methoxy-7-nitroindolinyl-caged-L-glutamate (i.e., MNI-caged glutamate; 250 μM; Tocris) added to recirculating, oxygenated ACSF. Brief pulses of fluorescent light (30 ms; UV filter; Chroma Technology) were directed into the slice through the 40× microscope objective with aperture and filter at lowest levels (Bhaskaran and Smith, 2010; Hunt et al., 2010, 2011). The objective was initially positioned to uncage glutamate directly over the recorded neuron, which consistently resulted in a large inward current and action potentials. The effective radius of stimulation (∼60 μm) was determined by manually moving the focal point of the stimulus in three different directions away from the recorded neuron until a direct inward current after stimulation was no longer observed. The objective was then moved to the dentate gyrus or CA3 pyramidal cell layer and photostimulation was applied focally to stereotyped sites along the DGC layer (tips of upper and lower blades, apex, and a midpoint on each blade between tip and apex) and in the proximal CA3 region. Five stimuli were applied per stimulation site at 0.1 Hz (five sweeps). For analysis, eEPSCs were assessed by comparing EPSC frequency during the period 1 s before (normalized to a 200-ms period) and 200 ms after the uncaging event (eEPSC frequency per sweep = #EPSCs after stim - #EPSCs prestim; final values were calculated as the mean difference over the five sweeps). Threshold for a positive responding site was set at a mean net increase of one eEPSC over five sweeps, as previously described (Hunt et al., 2011). Each eGFP+ neuron then received a percentage score for the number of effective stimulation sites after DGC and CA3 photostimulation (i.e., percentage stimulation sites resulting in a synaptic response).

We tested the possibility that variability in background sEPSC frequency could account for apparent synaptic responses; we analyzed seven to eight randomly selected 1200-ms segments of sEPSC recordings from eGFP+ neurons (i.e., in the absence of glutamate uncaging; *n* = 4–6 cells from each experimental group). Five sequential segments of recording were analyzed and averaged to mirror the five sweeps used in the analysis above. In each recording segment, the frequency of sEPSCs was measured during 1 s (normalized to a 200-ms bin) and then again in the subsequent 200 ms. sEPSC frequency in the subsequent 200 ms was then subtracted from the sEPSC frequency in the earlier normalized 200-ms bin to calculate a change in sEPSC frequency (i.e., ΔsEPSC frequency), analogous to how eEPSC frequency was calculated above (except no stimulus was applied). The mean ΔsEPSC frequency across all the experimental groups was 0.005 ± 0.032; only three of 192 (1.6%) ΔsEPSC frequencies were ≥1. The experimental groups did not statistically differ from one another in ΔsEPSC frequency values (Kruskal Wallis stat = 5.437, *p* = 0.3650 ^a^). These results indicate that eEPSC frequencies ≥1 are unlikely to be due to changes in background sEPSCs and supports the use of this threshold for defining positive stimulation sites.

### Statistical analysis

All data were assessed for normality using Shapiro-Wilk test and inspection of descriptive statistics to determine use of parametric or nonparametric statistical tests. Statistical analysis was performed using GraphPad Prism software (GraphPad Software), and a priori power analyses were used to determine sample sizes based on effect sizes from preliminary experiments. Parametric data were assessed using one-way ANOVA with Tukey’s *post hoc* comparisons. Nonparametric data were assessed using Kruskal-Wallis statistic with Dunn’s *post hoc* comparisons. Data are expressed as mean ± SEM, except that glutamate photostimulation data are expressed as the percentage of responding sites per neuron. Significance was set at *p* < 0.05. Table 1 indicates the tests used for each assessment and includes the retrospective power calculation for each statistical measurement.

**Table 1.** Statistical table

Outcome measure	Data structure	Type of test	Power
a. ΔsEPSC frequency	Nominal data, non-normal distribution	Kruskal Wallis	0.05
b. Weight change	Normal distribution	One-way ANOVA	0.99
c. Action potential firing rate	Normal distribution	One-way ANOVA	0.98
d. sEPSC frequency	Normal distribution	One-way ANOVA	0.8
e. sEPSC amplitude	Normal distribution	One-way ANOVA	0.06
f. eGFP+ neuron whole-cell capacitance	Normal distribution	One-way ANOVA	0.72
g. DGC RMP	Normal distribution	One-way ANOVA	0.05
h. eGFP+ neuron RMP	Normal distribution	One-way ANOVA	0.06
i. CA3 neuron RMP	Normal distribution	One-way ANOVA	0.11
j. Direct photostimulation evoked AP’s in DGCs	Normal distribution	One-way ANOVA	0.05
k. Direct photostimulation evoked AP’s in eGFP+ neurons	Normal distribution	One-way ANOVA	0.05
l. Direct photostimulation evoked AP’s in CA3 neurons	Normal distribution	One-way ANOVA	0.05
m. % effective stimulation sites to DG photostimulation per eGFP+ neuron, controls	Nominal data, non-normal distribution	Kruskal Wallis	0.05
n. % effective stimulation sites to DG photostimulation per eGFP+ neuron	Nominal data, non-normal distribution	Kruskal Wallis	0.68
o. % effective stimulation sites to CA3 photostimulation per eGFP+ neuron, controls	Nominal data, non-normal distribution	Kruskal Wallis	0.05
p. % effective stimulation sites to CA3 photostimulation per eGFP+ neuron	Nominal data, non-normal distribution	Kruskal Wallis	0.4
q. % effective stimulation sites to CA3 photostimulation per DGC nACSF	Nominal data, non-normal distribution	Kruskal Wallis	0.05
r. % responsive sites to CA3 photostimulation per DGC ACSF w/30 μM Bic	Nominal data, non-normal distribution	Kruskal Wallis	0.12

## Results

### Weight gain following CCI injury

A recent study in a mouse model of pilocarpine-induced SE described pathologic weight gain after SE that was reversed by rapamycin treatment (Hester et al., 2016). Additionally, rapamycin treatment in other murine models of disease has resulted in reduced weight gain and increased longevity (Deblon et al., 2012; Staats et al., 2013; Hebert et al., 2014; Hsieh et al., 2016). To determine the effect of CCI injury and rapamycin treatment on body weight, mice were weighed at the time of injury and at the time of tissue sample collection (8–12 weeks after injury) in a subset of the mice used in this study. There was a significant difference in weight gain between the three experimental groups (one-way ANOVA; *F*_(2,63)_ = 37.40; *p* < 0.0001^b^). Weight gain in vehicle-treated mice given sham or CCI injury was not significantly different (sham = 28.46 ± 2.94%; CCI = 22.92% ± 1.95%; Tukey’s: *p* = 0.9155), but mice given rapamycin treatment after CCI injury gained less weight than either sham or CCI injured mice (CCI + Rapa = 7.61 ± 0.89%; Tukey’s: vs sham *p* < 0.0001; vs CCI *p* < 0.0001). Neither pathologic weight gain following CCI injury, as reported following pilocarpine-induced SE (Hester et al., 2016), nor weight loss after rapamycin were observed. Instead, rapamycin treatment resulted in reduced weight gain following CCI injury, similar to previous reports (Deblon et al., 2012; Staats et al., 2013; Hebert et al., 2014; Hsieh et al., 2016).

### Spontaneous action potential and sEPSC frequency in eGFP+ hilar interneurons

**Figure 1.**
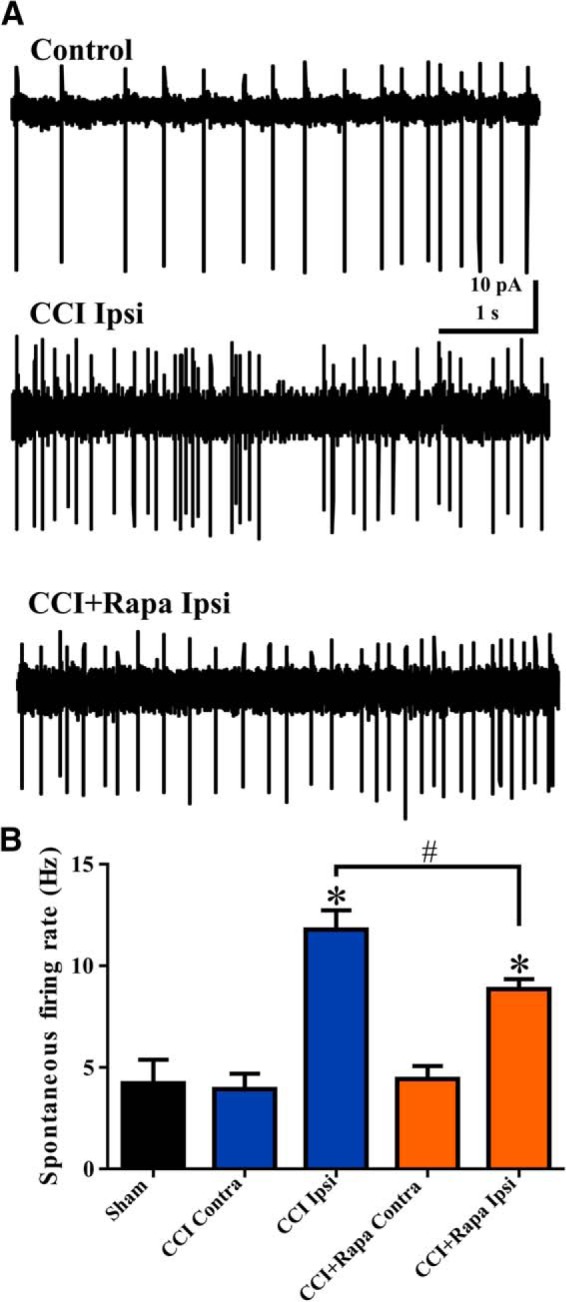
Rapamycin treatment reduces, but does not normalize, the increased activity of hilar inhibitory interneuron 8–12 weeks after CCI injury. ***A***, Representative traces showing spontaneous action potential firing from three different treatment groups: control (i.e., sham and contralateral neurons), ipsilateral to CCI injury + vehicle (CCI Ipsi), and ipsilateral to CCI injury + 3 mg/kg rapamycin (CCI + Rapa Ipsi). ***B***, Mean spontaneous action potential firing in sham, CCI Contra, CCI Ipsi, CCI + Rapa Contra, and CCI + Rapa Ipsi groups. Error bars indicate SEM; *p < 0.05 compared to sham and contralateral hemispheres; #*p* < 0.05 for CCI Ipsi versus CCI + Rapa Ipsi.

Weeks after pilocarpine-induced SE or CCI injury, surviving eGFP+ hilar interneurons ipsilateral to the injury display increased spontaneous action potential firing and sEPSC frequency (Halabisky et al., 2010; Hunt et al., 2011). Rapamycin treatment suppresses MFS in the dentate gyrus (Buckmaster and Lew, 2011; Guo et al., 2013; Butler et al., 2015), but the effect of mTOR inhibition on activity and synaptic input to eGFP+ hilar interneurons after CCI is not known. Spontaneous action potential firing rate was recorded (on cell) in eGFP+ hilar interneurons in slices from sham-injured mice (*n* = 6 cells, 5 mice), CCI-injured mice that were treated daily with vehicle (contralateral, *n* = 10; ipsilateral, *n* = 12; 10 mice), and CCI-injured mice that were treated daily for 8–12 weeks with rapamycin (contralateral, *n* = 8; ipsilateral, *n* = 9; 11 mice). There was no significant difference between cells from contralateral and ipsilateral hemispheres in sham-injured mice for any measurements made (*p* > 0.05); results from sham-injured mice were therefore combined into one group. Representative traces showing spontaneous action potential firing in eGFP+ neurons from control (i.e., sham and contralateral hemisphere), ipsilateral to CCI injury with vehicle treatment, and ipsilateral to CCI injury with rapamycin treatment groups are shown in Figure 1*A*. We detected increased firing rates in eGFP+ hilar interneurons ipsilateral to CCI injury relative to eGFP+ interneurons from the contralateral hippocampus or from sham-injured mice, as has been reported previously (Hunt et al., 2011; sham = 4.22 ± 1.16 Hz; CCI contra = 3.93 ± 0.76 Hz; CCI ipsi = 11.79 ± 0.94 Hz; *F*_(4,43)_ = 21.38; *p* < 0.0001^c^, Tukey’s: sham vs CCI ipsi *p* = 0.0002; CCI contra vs CCI ipsi *p* < 0.0001; Fig. 1*B*). After rapamycin treatment, action potential firing rate in eGFP+ interneurons ipsilateral to CCI-injury was reduced relative to CCI + vehicle mice, but remained significantly elevated compared to eGFP+ interneurons from sham-injured mice or cells contralateral to CCI injury (CCI + Rapa contra = 4.43 ± 0.64 Hz, CCI + Rapa ipsi = 8.86 ± 0.49 Hz, Tukey’s: sham vs CCI + Rapa ipsi *p* < 0.0001; CCI contra vs CCI + Rapa ipsi *p* = 0.0002; CCI + Rapa contra vs CCI + Rapa ipsi *p* = 0.0003; CCI ipsi vs CCI + Rapa ipsi *p* = 0.014; Fig. 1*B*). The effect of ionotropic glutamate receptor blockade on spontaneous action potential firing was determined in a subset of eGFP+ hilar interneurons. Addition of kynurenic acid (30 μM) significantly reduced action potential frequency by >50% in all experimental groups (paired *t* test; *t*_(13)_ = 5.108; *p* = 0.0002). Synaptic input therefore contributed significantly to action potential frequency, and rapamycin treatment reduced, but did not normalize action potential firing in eGFP+ hilar interneurons ipsilateral to CCI injury. Further investigation therefore focused on the effect of rapamycin treatment on the reorganization of glutamatergic synaptic input to surviving hilar eGFP+ neurons.

**Figure 2.**
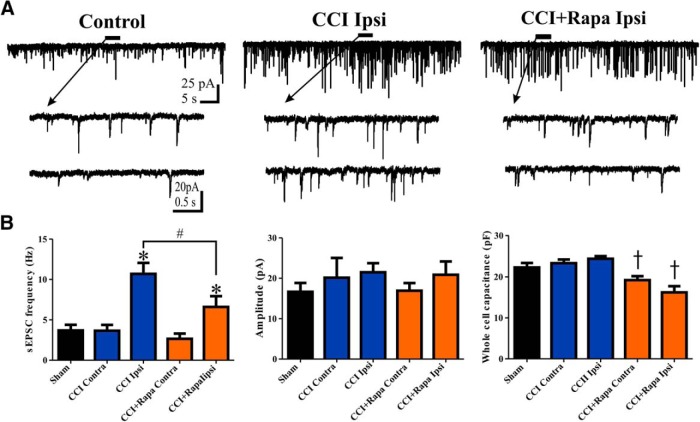
Rapamycin treatment reduces, but does not normalize, the increase in sEPSC frequency in hilar inhibitory interneurons 8–12 weeks after CCI injury. ***A***, Representative traces showing sEPSCs in eGFP+ neurons from three different treatment groups: control (i.e., sham and contralateral neurons), ipsilateral to CCI injury + vehicle (CCI Ipsi), and ipsilateral to CCI injury + 3 mg/kg rapamycin (CCI + Rapa Ipsi). Expanded sections of the trace under the black line are indicated by arrows. ***B***, Mean sEPSC frequency, amplitude, and whole-cell capacitance in sham, CCI Contra, CCI Ipsi, CCI + Rapa Contra, and CCI + Rapa Ipsi groups. Error bars indicate SEM; *p < 0.05 compared to sham and contralateral hemispheres; #*p* < 0.05 for CCI Ipsi versus CCI + Rapa Ipsi; †*p* < 0.05 compared to sham, CCI Contra, and CCI Ipsi.

The sEPSC frequency in eGFP+ hilar interneurons was determined using whole-cell patch-clamp recordings. Representative traces showing sEPSCs in eGFP+ cells from control (i.e., sham and contralateral hemisphere), ipsilateral to CCI injury with vehicle treatment, and ipsilateral to CCI injury with rapamycin treatment groups are shown in Figure 2*A*. There was an increased sEPSC frequency in eGFP+ hilar interneurons ipsilateral to CCI relative to sham or contralateral to CCI, as reported previously (Hunt et al., 2011; sham: 3.69 ± 0.71 Hz; CCI contra = 3.65 ± 0.73 Hz; CCI ipsi = 10.69 ± 1.36 Hz; *F*_(4,45)_ = 9.478; *p* < 0.0001^d^; Tukey’s: sham vs CCI ipsi *p* = 0.0034; CCI contra vs CCI ipsi *p* = 0.0003; Fig. 2*B*). Rapamycin treatment significantly reduced, but did not normalize, the increased sEPSC frequency in ipsilateral eGFP+ neurons (CCI + Rapa contra = 2.66 ± 0.65 Hz; CCI + Rapa ipsi = 6.60 ± 1.33 Hz; Tukey’s: sham vs CCI + Rapa ipsi *p* = 0.0182; CCI contra vs CCI + Rapa ipsi *p* = 0.0356; CCI + Rapa contra vs CCI + Rapa ipsi *p* = 0.0127; CCI ipsi vs CCI + Rapa ipsi *p* = 0.014; Fig. 2*B*). These results indicate that rapamycin treatment after CCI reduced, but did not normalize, the increase in sEPSC frequency in eGFP+ interneurons ipsilateral to CCI injury. We therefore hypothesized that rapamycin affected components of the reorganization of synaptic input to surviving hilar eGFP+ neurons after brain injury.

To assess the influence of active CA3 pyramidal neurons on glutamatergic synaptic input to surviving hilar eGFP+ neurons, we microdissected the CA3 pyramidal cell layer from the hilus and dentate gyrus. In the isolated dentate gyrus/hilus slice, sEPSC frequency in eGFP+ hilar neurons ipsilateral to CCI injury remained elevated relative to sham and contralateral control neurons (control = 3.84 ± 0.65 Hz, *n* = 20; CCI ipsi = 10.37 ± 1.56 Hz, *n* = 12; one-way ANOVA; *F*_(2,39)_ = 8.844; *p* = 0.0007; Tukey’s control vs CCI ipsi *p* = 0.0005), and sEPSC frequency in mice that received rapamycin treatment was not different from control neurons (CCI + Rapa ipsi = 7.12 ± 1.62 Hz, *n* = 10; Tukey’s control vs CCI + Rapa ipsi *p* = 0.13). Removing CA3 did not significantly alter sEPSC frequency in any group (*p* > 0.05). Because removing CA3 did not alter the sEPSC frequency differences between groups, and previous findings indicated that action potential-independent glutamate release in GFP+ hilar interneurons was elevated after CCI (Hunt et al., 2011), we investigated the effect of rapamycin on excitatory synaptic input arising from CA3 pyramidal cells and DGCs.

### sEPSC amplitude and whole-cell capacitance in eGFP+ hilar interneurons

The amplitude of unitary sEPSCs in eGFP+ hilar interneurons has been shown to be unaltered in CCI injured mice compared to the contralateral hemisphere and controls (Hunt et al., 2011). To assess the effect of rapamycin on sEPSC amplitude, the amplitude of unitary sEPSCs was compared in sham and CCI injured mice with vehicle or rapamycin treatment. There was no difference in amplitude of sEPSCs between experimental groups (sham = 16.65 ± 2.18 pA; CCI contra = 20.14 ± 4.89 pA; CCI ipsi = 21.49 ± 2.25 pA; CCI + Rapa contra = 16.90 ± 1.91 pA; CCI + Rapa ipsi = 20.86 ± 3.30 pA; one-way ANOVA; *F*_(4,45)_ = 0.6436; *p* = 0.7413^e^; Fig. 2*B*). These results suggested that effects of rapamycin on sEPSC and action potential firing frequency were not due to postsynaptic changes in glutamate receptors in eGFP+ hilar interneurons.

In the pilocarpine-induced SE model of TLE, an increase in soma size of surviving hilar interneurons has also been reported, and rapamycin treatment reduced the increase in soma size associated with TLE development (Buckmaster and Wen, 2011). As an indirect assessment of soma size, whole-cell capacitance was measured from recorded eGFP+ hilar interneurons to assess effects of CCI and rapamycin treatment. An overall effect of treatment was detected, but CCI injury did not increase whole-cell capacitance of eGFP+ hilar interneurons in the ipsilateral hemisphere (sham = 22.31 ± 1.09 pF; CCI contra = 23.36 ± 0.89 pF; CCI ipsi = 24.44 ± 0.65 pF; *F*_(4,46)_ = 8.187; *p* < 0.0001^f^; Tukey’s: sham vs CCI contra *p* = 0.4656; sham vs CCI ipsi *p* = 0.1201; CCI contra vs CCI ipsi *p* = 0.3576; Fig. 2*B*). Rapamycin treatment after CCI reduced the whole-cell capacitance of these neurons relative to CCI alone or sham injured groups (CCI + Rapa contra = 19.22 ± 0.97 pF, CCI + Rapa ipsi = 16.21 ± 1.52 pF; Tukey’s: sham vs CCI + Rapa contra *p* = 0.0406; sham vs CCI + Rapa ipsi *p* = 0.0121; CCI contra vs CCI + Rapa contra *p* = 0.0089; CCI ipsi vs CCI + Rapa contra *p* = 0.0017; CCI ipsi vs CCI + Rapa ipsi *p* = 0.001; CCI contra vs CCI + Rapa ipsi *p* = 0.0023; Fig. 2*B*). These data suggest that while CCI injury did not alter cell size, rapamycin treatment reduced cell size in this hilar interneuron population.

### Rapamycin suppressed the injury-induced increase in synaptic responses of eGFP+ interneurons after glutamate photostimulation of DGCs

After CCI, surviving eGFP+ hilar interneurons ipsilateral to injury exhibit increased excitatory synaptic input arising from both DGCs and CA3 pyramidal neurons (Hunt et al., 2011). mTOR inhibition reduces MFS and recurrent excitation after chemoconvulsant-induced SE or CCI, and also reduces the number of excitatory synapses per DGC in the inner molecular layer after SE (Yamawaki et al., 2015). In addition to these structural data, our functional measures of spontaneous synaptic excitation and action potential firing in eGFP+ hilar interneurons prompted us to hypothesize that mTOR inhibition would inhibit synaptic reorganization of DGC or CA3 input to hilar interneurons after CCI. Here, recordings were performed from eGFP+ hilar interneurons during glutamate photostimulation of either the DGC layer or CA3 to assess the effect of continuous rapamycin treatment on injury-induced synaptic changes arising from these sites.

Before testing synaptic connectivity, both responses to direct activation by glutamate uncaging (i.e., action potential firing) and resting membrane potential (RMP) were measured in the three cell types (DGCs, eGFP+ hilar interneurons, and CA3 pyramidal neurons) to determine the effect of injury and drug treatment on these intrinsic properties (Table 2). In each cell type, neither injury nor rapamycin treatment had a significant effect on RMP (DGC: *F*_(2,85)_ = 0.1923, *p* = 0.8254^g^; eGFP+ interneuron: *F*_(2,35)_ = 1.312, *p* = 0.2822^h^; CA3 pyramidal neuron: *F*_(2,26)_ = 2.660, *p* = 0.0889^i^). Similarly, no effect of injury or rapamycin was detected for direct responses to glutamate uncaging (DGC: *F*_(2,85)_ = 0.3683, *p* = 0.6928^j^; eGFP+ interneuron: *F*_(2,35)_ = 0.1197, *p* = 0.8876^k^; CA3 pyramidal neuron: *F*_(2,26)_ = 0.1595, *p* = 0.8534^l^). This is consistent with previous results showing similar responses of eGFP+ hilar neurons and CA3 pyramidal cells to direct glutamate photostimulation after CCI injury (Hunt et al., 2011). Thus, RMP values and responses to direct glutamate photostimulation (i.e., action potential number) were comparable between groups, suggesting that changes in the intrinsic properties of these cells did not have a large impact on our subsequent glutamate uncaging measures to assess synaptic connectivity.

**Table 2.** RMP and direct photoactivation measures for DGCs, eGFP hilar neurons, and CA3 pyramidal neurons

Cell type	Group	Number of cells	Number of animals	Direct photoactivation (number of APs)	RMP (mV)
DGC	Control	66	32	5.31 ± 0.43	−67.11 ± 0.97
DGC	CCI ipsi	24	7	6.04 ± 0.80	−66.73 ± 2.18
DGC	CCI + Rapa ipsi	14	7	5.54 ± 0.92	−65.63 ± 2.03
eGFP+ neuron	Control	20	15	3.88 ± 0.57	−54.72 ± 1.95
eGFP+ neuron	CCI ipsi	10	6	3.44 ± 0.62	−52.33 ± 1.63
eGFP+ neuron	CCI + Rapa ipsi	8	7	3.78 ± 0.72	−60.64 ± 3.46
CA3 neuron	Control	16	11	3.79 ± 0.35	−55.76 ± 1.78
CA3 neuron	CCI ipsi	7	4	4.03 ± 0.76	−49.53 ± 2.77
CA3 neuron	CCI + Rapa ipsi	6	3	3.53 ± 0.61	−48.39 ± 4.23

No significant differences were detected within any cell type.

**Figure 3.**
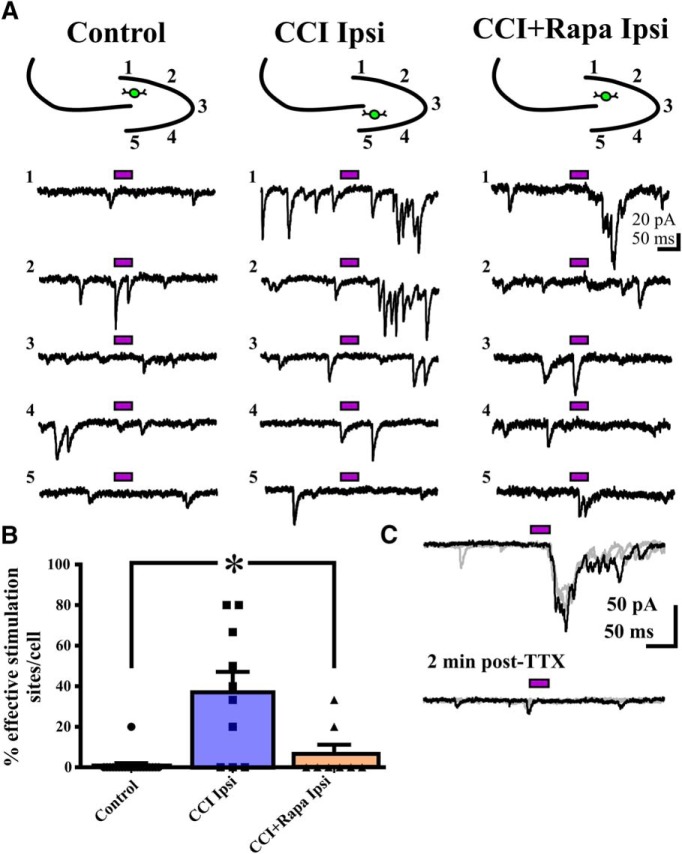
Rapamycin treatment abrogates the injury-induced increase in synaptic input from DGCs to hilar inhibitory interneurons 8–12 weeks after CCI injury. ***A***, Representative eEPSC responses in eGFP+ interneurons to glutamate photostimulation applied to DGCs from three different treatment groups: control (i.e., sham and contralateral hemispheres), CCI Ipsi, and CCI + Rapa Ipsi. Bars above traces indicate glutamate photostimulation period. The relative position of the recorded hilar interneuron (green) and numbered stimulation sites in the dentate gyrus are shown on the stereotyped drawing above each set of traces; numbers for traces correspond to stimulation site numbers in the drawing. ***B***, Individual and mean percentage of effective stimulation sites in control, CCI Ipsi, and CCI + Rapa Ipsi groups. ***C***, The synaptic response of a eGFP+ hilar interneuron after photostimulation of DGCs (top trace) is blocked in the presence of TTX (2 µM; bottom trace), additional stimulation sweeps in gray. Error bars indicate SEM; *p < 0.05 compared to control. The number of stimulation sites, cells, and animals from separate treatment groups are presented in Table 3.

Glutamate uncaging was then performed within the DGC layer while recording synaptic responses within eGFP+ hilar interneurons. Figure 3*A* shows representative traces of synaptic responses of eGFP+ hilar interneurons to glutamate photostimulation of DGCs for control (i.e., sham and vehicle/rapamycin-treated contralateral hemispheres), ipsilateral to CCI injury with vehicle treatment, and ipsilateral to CCI injury with rapamycin treatment groups. A similar analysis to that of Hunt et al. (2011) was performed to test for positive responses to glutamate photolysis, while accounting for background sEPSCs during each recording. A photostimulation site was considered to evoke a positive response if it resulted in a mean eEPSC frequency ≥1 within a recorded eGFP+ hilar interneuron (see Materials and Methods). Subsequently, each recorded neuron received a score based on the percentage of positive stimulation sites within the DGC layer. Responses of eGFP+ neurons to glutamate photostimulation of DGCs were tetrodotoxin (TTX)-sensitive in 5/5 (100%) of cells tested (Fig. 3*C*), indicating the synaptic responses originated subsequent to action potential generation in photostimulated neurons.

The number of stimulation sites, cells, and animals from separate treatment groups (sham-injured animals and the contralateral and ipsilateral hemisphere of injured animals given either vehicle or rapamycin treatment) are presented in Table 3. No significant difference in the percentage of effective stimulation sites to DGC photostimulation was detected between eGFP+ hilar interneurons located contralateral to injury (CCI + vehicle or CCI + rapamycin treatment) or from sham-injured animals (Kruskal Wallis stat = 1.004, *p* = 0.6053^m^; Table 3). Therefore, all eGFP+ hilar interneuron recordings from sham injury or contralateral to injury were combined into a single control group for clarity of analysis and presentation. There was a significant overall difference in the percentage of responsive DGC photostimulation sites between recordings from controls and those from the ipsilateral hemisphere of CCI-injured animals administered either vehicle or rapamycin (Kruskal-Wallis stat = 16.08, *p* = 0.0003^n^; Fig. 3). Interneurons ipsilateral to CCI injury with vehicle treatment exhibited a significantly greater percentage of effective stimulation sites per neuron compared to controls (control = 1 ± 1.00%, CCI ipsi = 37.00 ± 10.09%; Dunn’s: *p* = 0.0002; Fig. 3). Rapamycin treatment significantly reduced the percentage of sites responsive to DGC photostimulation, such that eGFP+ interneurons from mice given CCI + rapamycin treatment were indistinguishable from controls (CCI + Rapa ipsi = 6.67 ± 4.54%; Dunn’s: vs control *p* > 0.9999; vs CCI ipsi *p* = 0.0455; Fig. 3). Therefore, rapamycin treatment abrogated the injury-induced increase in excitatory synaptic input to eGFP+ interneurons arising from glutamate uncaging within the DGC layer.

**Table 3.** Responses of eGFP+ hilar inhibitory interneurons to photostimulation of DGCs and CA3 neurons

Group	Responsive DG stimulation sites	Responsive CA3 stimulation sites	Number of cells	Number of animals	Net eEPSC frequency DG stimulatio n	Net eEPSC frequency CA3 stimulation
Sham	1/34	0/18	9	5	0.13 ± 0.08	0.09 ± 0.10
CCI contra	3/36	1/14	6	5	0.31 ± 0.13	0.28 ± 0.14
CCI ipsi	22/55*	10/25*	10	6	0.90 ± 0.19*	1.31 ± 0.43*
CCI + Rapa contra	1/26	1/10	5	5	0.04 ± 0.10	0.02 ± 0.38
CCI + Rapa ipsi	7/40	8/18*	8	7	0.33 ± 0.11	1.27 ± 0.44*

Significant differences from control indicated with an asterisk.

### Rapamycin failed to suppress the injury-induced increase in synaptic responses of eGFP+ interneurons to photoactivation of CA3 pyramidal cells

**Figure 4.**
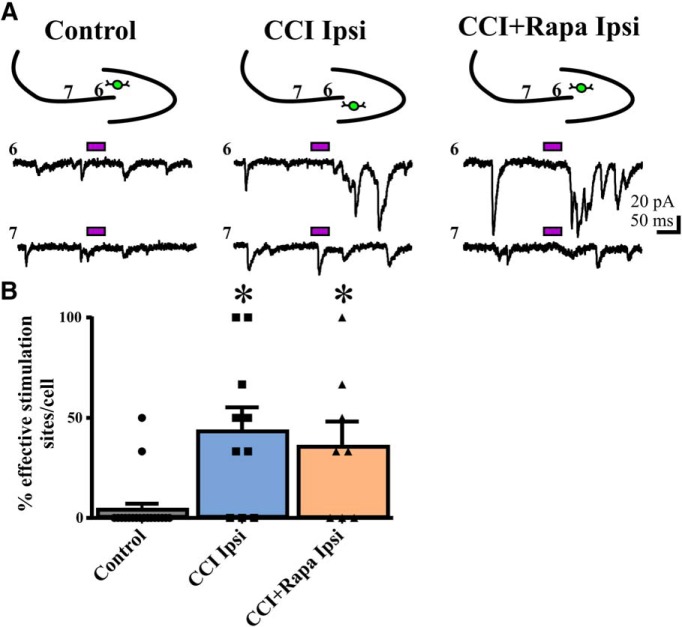
Rapamycin fails to suppress the injury-induced increase in eEPSC responses from CA3 pyramidal cells to eGFP+ hilar interneurons 8–12 weeks after injury. ***A***, Representative traces showing responses in eGFP+ hilar interneurons to glutamate photostimulation applied to CA3 pyramids from three different treatment groups: control, CCI Ipsi, and CCI + Rapa Ipsi. Bars above traces indicate glutamate photostimulation period. The relative position of the recorded hilar interneuron (green) and numbered stimulation sites in the CA3 pyramidal cell layer are shown on the stereotyped drawing above each set of traces. ***B***, Individual and mean percentage of effective stimulation sites in control, CCI Ipsi, and CCI + Rapa Ipsi groups. Error bars indicate SEM; *p < 0.05 compared to control. The number of stimulation sites, cells, and animals from separate treatment groups are presented in Table 3.

After CCI, CA3 pyramidal cells form new functional connections with hilar inhibitory interneurons (Hunt et al., 2011). In addition to photostimulation of DGCs, we also measured responses of eGFP+ hilar interneurons to photostimulation of CA3 pyramidal cells. We again defined positive synaptic response sites as sites that resulted in a net eEPSC frequency ≥1 within recorded eGFP+ hilar interneurons. Each recorded neuron then received a score based on the percentage of positive stimulation sites within the CA3 pyramidal neuron layer. Representative traces showing responses of eGFP+ hilar interneurons to glutamate photostimulation of CA3 pyramidal cells (Fig. 4*A*) are shown from control (i.e., sham and vehicle/rapamycin-treated contralateral hemispheres), ipsilateral to CCI injury with vehicle treatment, and ipsilateral to CCI injury with rapamycin treatment groups. The number of stimulation sites, cells, and animals from separate treatment groups are presented in Table 3. Similar to the photostimulation of DGCs, no significant difference in response to CA3 photostimulation was detected between sham-injured animals and those located contralateral to injury (CCI + vehicle or CCI + rapamycin treatment; Kruskal-Wallis stat = 0.8771, *p* = 0.6450^°^). Therefore, these data were combined into a single control group. For these controls, the average percentage of positive sites responding to CA3 photostimulation was 4.17 ± 2.93% (Fig. 4).

Similar to responses after DGC photostimulation, there was a significant overall increase in the percentage of responsive CA3 photostimulation sites between recordings from controls and those from the ipsilateral hemisphere of CCI-injured animals administered either vehicle or rapamycin (Kruskal-Wallis stat = 13.21, *p* = 0.0014^p^; Fig. 4). Interneurons from CCI-injured, vehicle-treated mice exhibited a significantly increased percentage of positive responding sites after CA3 pyramidal cell photostimulation relative to controls (CCI ipsi = 43.33 ± 11.97%; Dunn’s: vs control *p* = 0.0033; Fig. 4). Unlike for DGC photostimulation, rapamycin treatment did not significantly suppress the CCI-induced increase in synaptic input to eGFP+ interneurons arising from CA3 pyramidal cells. Photostimulation of CA3 pyramidal cells resulted in a significantly larger percentage of photostimulation sites that evoked eEPSCs in eGFP+ hilar interneurons from the ipsilateral hemisphere of CCI + rapamycin treated animals relative to controls (CCI + Rapa ipsi = 35.42 ± 12.77%; Dunn’s: vs control *p* = 0.0345; Fig. 4). There was no significant difference in the percentage of CA3 effective stimulation sites between rapamycin- and vehicle-treated groups after CCI injury (Dunn’s vs CCI ipsi *p* > 0.9999). Collectively, these results indicate that rapamycin treatment reduces the excessive synaptic connections from DGCs to eGFP+ hilar interneurons after focal brain injury, but fails to suppress aberrant connections arising from CA3 pyramidal cells.

### Responses of DGCs to glutamate photostimulation of CA3 pyramidal neurons

Sparse synaptic connections from CA3 pyramidal neurons to DGCs have been described previously in naïve rats (Scharfman, 1994a,b, 2007), and this “backprojection” may contribute to hyperexcitability of DGCs through either monosynaptic or polysynaptic connections in models of epilepsy (Scharfman, 1994a, Scharfman et al., 2001; Scharfman, 2007). Changes in the backprojection from CA3 neurons to DGCs following CCI injury, and the effect of rapamycin on this potential synaptic reorganization, have not been previously studied. However, projections from CA3 could contribute to the increase in excitatory synaptic drive to DGCs observed after CCI injury (Hunt et al., 2009, 2011; Butler et al., 2015). Glutamate photolysis was used to study the CA3-to-DGC backprojection using the same approach and analysis as performed to test synaptic input to hilar interneurons.

**Figure 5.**
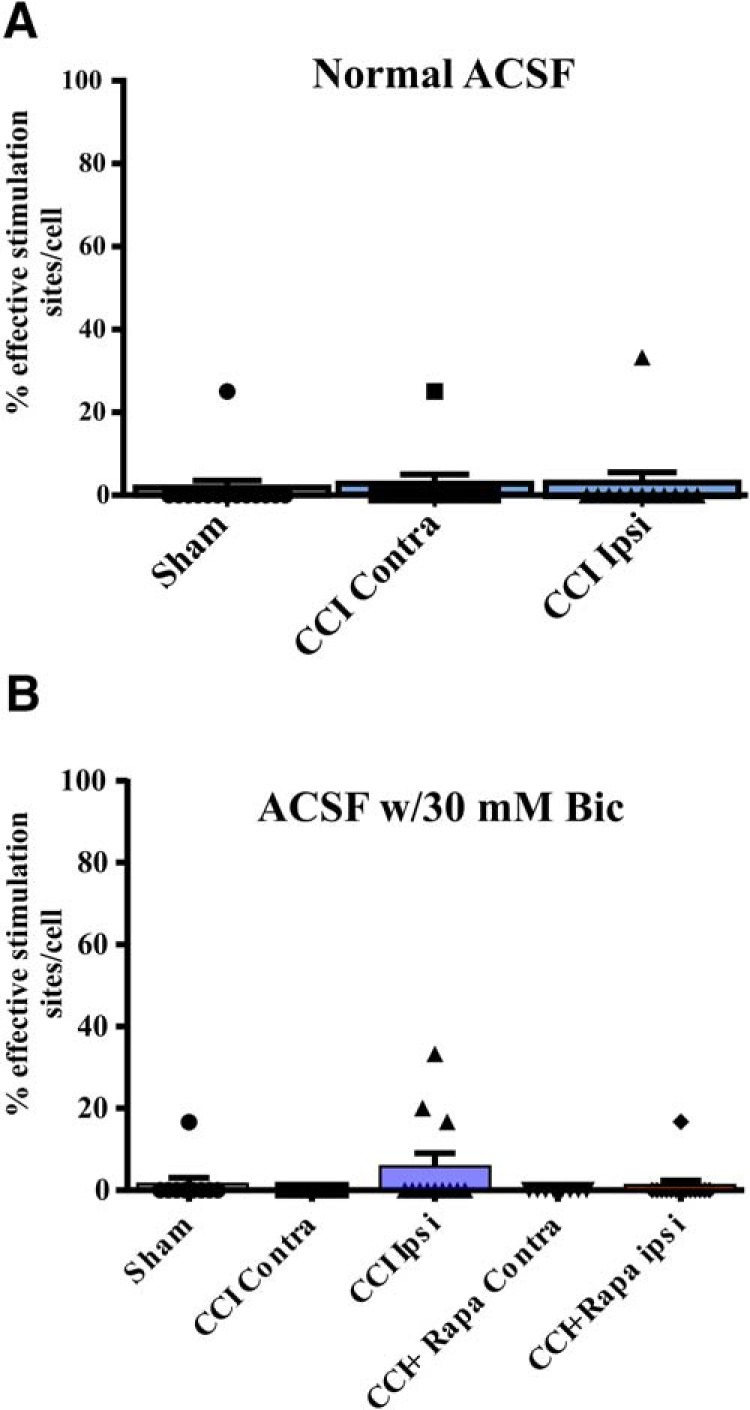
eEPSC responses in DGCs after glutamate photostimulation of CA3 pyramidal cells 8–12 weeks after injury. ***A***, Individual and mean percentage of effective stimulation sites. Responses in DGCs from three different treatment groups: sham, CCI Contra, and CCI Ipsi in normal ACSF. ***B***, Individual and mean percentage of effective stimulation sites for DGCs in the presence of 30 μM bicuculline from three different treatment groups: control (i.e., sham and contralateral hemispheres), CCI Contra and Ipsi, and CCI + Rapa Contra and Ipsi. The number of stimulation sites, cells, and animals from separate treatment groups are presented in Tables 4, 5.

**Figure 6.**
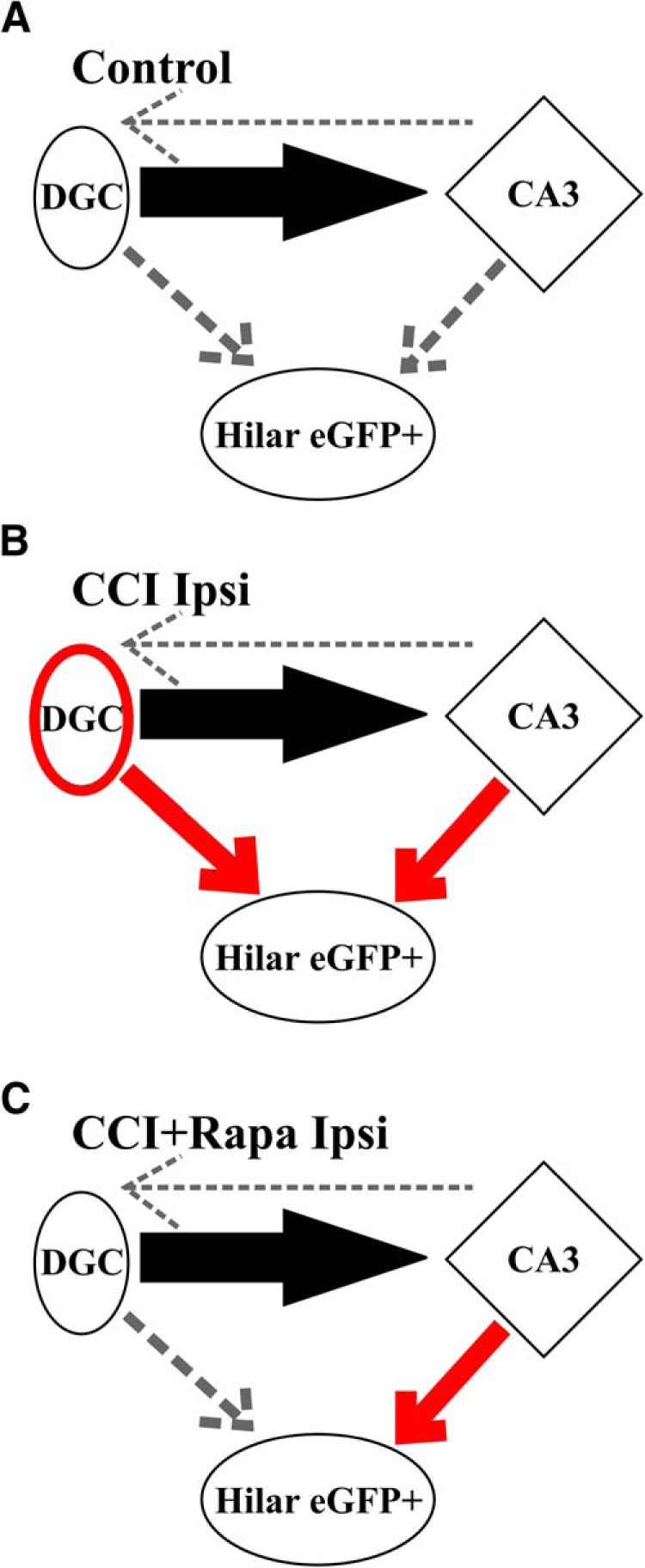
Diagramatic representation of effects of rapamycin treatment on dentate gyrus circuitry after focal brain injury. ***A***, Normal circuit in dentate gyrus. The projection of a DGC onto a CA3 pyramidal cell is shown (solid black arrow). GABAergic hilar inhibitory interneurons are also present, but are not robustly innervated by DGCs or CA3 pyramidal cells (dashed arrows). ***B***, Functional synaptic input to surviving hilar interneurons arising from activity in both DGCs and CA3 pyramidal cells (red arrows) are increased ipsilateral to CCI injury, as are connections between DGCs (red oval; Hunt et al., 2010, 2011; Butler et al., 2015). ***C***, mTOR inhibition after CCI injury reduces reorganization of functional DGC connections with surviving hilar inhibitory interneurons, but aberrant excitatory connection arising from CA3 pyramidal cell activity is sustained. This cartoon does not discriminate between mono- and polysynaptic connections. Line thickness is used as a surrogate marker for the percentage effective stimulation sites as assessed by glutamate photolysis here and in Hunt et al. (2010).

The number of stimulation sites, cells, and animals from separate treatment groups are presented in Tables 4, 5. Synaptic responses of DGCs to photostimulation of CA3 pyramidal cells were measured in normal ACSF or when GABA_A_ receptors were blocked to promote synaptic disinhibition. In normal ACSF, responses of DGCs to photostimulation of CA3 pyramidal cells were relatively sparse; there was no significant difference in the percentage of effective stimulation sites in CA3 that resulted in eEPSCs in DGCs between sham-treated mice and contralateral or ipsilateral to CCI injury after during photostimulation of CA3 pyramidal cells (sham = 1.79 ± 1.79%; CCI contra = 2.50 ± 2.50%; CCI ipsi = 2.78 ± 2.78%: Kruskal-Wallis stat = 0.0621, *p* = 0.9694^q^; Table 4; Fig. 5*A*). To unmask potential polysynaptic excitatory connections, bicuculline (30 μM) was added to the ACSF and effective stimulation sites in CA3 were again tested in sham-injured mice and in vehicle- or rapamycin-treated, CCI-injured mice. In the presence of bicuculline, photostimulation of CA3 again resulted in only sparse positive responses in DGCs and there was no significant difference between DGCs from sham-injured mice or from the contralateral or ipsilateral hemispheres of CCI-injured mice given vehicle or rapamycin (sham = 1.52 ± 1.52%, CCI contra = 0.0 ± 0.0%, CCI ipsi = 5.83 ± 3.23%, CCI + Rapa contra = 0.0 ± 0.0%, CCI + Rapa ipsi = 1.19 ± 1.19%; Kruskal-Wallis stat = 6.165; *p* = 0.1872^r^; Table 5; Fig. 5*B*). Thus, activation of CA3 pyramidal cells rarely resulted in increased synaptic activation of DGCs, regardless of injury or rapamycin treatment. The effects of mTOR inhibition on excitatory synaptic reorganization onto surviving hilar inhibitory interneurons after CCI injury are summarized in Figure 6.

**Table 4.** Responses of DGCs to photostimulation of CA3 neurons in normal ACSF

Group	Number of effective stimulation sites	Number of cells	Number of animals
Sham	1/67	14	4
CCI contra	1/46	10	6
CCI ipsi	3/57	12	5

**Table 5.** Responses of DGCs from control groups to photostimulation of CA3 neurons in ACSF containing 30 μM bicuculline

Group	Number of effective stimulation sites	Number of cells	Number of animals	Net eEPSC frequency
Sham	2/57	11	4	0.22 ± 0.06
CCI contra	0/40	9	6	0.13 ± 0.06
CCI ipsi	3/49	12	6	0.08 ± 0.04
CCI + Rapa contra	0/49	10	7	0.05 ± 0.04
CCI + Rapa ipsi	1/78	14	7	0.01 ± 0.01

## Discussion

Rapamycin treatment abrogates the synaptic reorganization among DGCs that occurs after SE or TBI (Buckmaster et al., 2009; Zeng et al., 2009; Buckmaster and Lew, 2011; Guo et al., 2013; Heng et al., 2013; Butler et al., 2015). However, the correlation of rapamycin’s effect on this synaptic reorganization with epileptogenesis varies across animal models. By two months after CCI injury, spontaneous seizures and a number of cellular correlates of posttraumatic epileptogenesis emerge in 40–50% of mice (Hunt et al., 2009, 2010; Guo et al., 2013). Rapamycin treatment after CCI injury reduces seizure prevalence and MFS, but does not block other cellular changes associated with epileptogenesis (Guo et al., 2013; Butler et al., 2015). After pilocarpine-induced SE, rapamycin treatment does not attenuate seizures, even at doses that block nearly all MFS (Buckmaster and Lew, 2011; Heng et al., 2013), but mTOR inhibition after kainic acid-induced SE does reduce seizure frequency (Zeng et al., 2009). It is important to understand the mechanistic effects of rapamycin because it is currently in use, or is in consideration for use, as a treatment cancer, tubular sclerosis and developmental malformations. The principal findings here indicate that mTOR inhibition reduces, but does not eliminate, the postinjury increase in excitability of hilar inhibitory interneurons by inhibiting their downstream responses during activation of DGCs, without suppressing their downstream responses during activation of CA3 pyramidal cells. These results are consistent with a rapamycin-dependent blockade of axonal sprouting of DGCs in combination with a lack of blocking effect on axonal sprouting of CA3 pyramidal cells (i.e., the CA3 backprojection) to these interneurons. This result implies that there are multiple mechanistic pathways underlying hippocampal synaptic reorganization after brain injury and highlights the challenges of preventing reactive plasticity associated with epileptogenesis.

This study investigated the effect of mTOR inhibition after CCI injury on the reorganization of synaptic input to a subset of hilar interneurons that are significantly depleted in models of TBI and TLE, even when mTOR is inhibited (Lowenstein et al., 1992; Buckmaster and Wen, 2011; Butler et al., 2016). Functionally, these interneurons contribute to shunting of synaptic excitation through the DGC layer, and the loss of these neurons could contribute to increased seizure susceptibility (Buckmaster and Dudek, 1997; Buckmaster and Wen, 2011; Hunt et al., 2011). Synaptic reorganization originating from DGCs and CA3 pyramidal cells onto surviving hilar interneurons may partially compensate for the loss of these cells after brain injury. Conversely, hyperactivity of surviving inhibitory interneurons could maintain a state of imbalanced synaptic and nonsynaptic GABAergic signaling to DGCs (Boychuk et al., 2016), thereby degrading the overall stability of dentate gyrus circuitry. Coordinated activity in synaptically connected inhibitory neurons can also synchronize activity in their targets, possibly due to rebound excitation, and hyperexcitability of these cells may promote epileptiform activity (Wendling et al., 2002; Bonifazi et al., 2009; Yekhlef et al., 2015; Shiri et al., 2017). In this case, the failure of rapamycin treatment to reduce the increased synaptic excitation of inhibitory interneurons arising from CA3 could increase the propensity for coordinated activity in DGCs and maintain an underlying excitability increase that is expressed as a reemergence of epileptogenesis when rapamycin treatment is removed.

Ipsilateral to injury, spontaneous action potential firing and sEPSC frequency in surviving hilar interneurons were increased 8-12 weeks after CCI, consistent with previous reports in models of TLE and PTE (Halabisky et al., 2010; Hunt et al., 2011). Blocking ionotropic glutamate receptors with kynurenic acid significantly reduced action potential firing, implying a major role for excitatory synaptic transmission in regulating activity of eGFP+ hilar interneurons. We found that the injury-induced increase in excitability of ipsilateral eGFP+ hilar interneurons was reduced after rapamycin treatment, but not normalized to control levels, with no change in the hemisphere contralateral to injury. There was no injury- or rapamycin-induced change in sEPSC amplitudes, responses to direct glutamate photostimulation, or RMP in any cell type recorded, suggesting that postsynaptic changes in glutamate responsiveness and intrinsic properties likely did not account for the increase in evoked synaptic responses. Synaptic responses to photostimulation of DGCs or CA3 cells were blocked by TTX, consistent with involvement of intact, functional synaptic connections originating in the activated cells.

Daily rapamycin treatment in models of PTE (Guo et al., 2013; Butler et al., 2015) and TLE (Buckmaster et al., 2009; Zeng et al., 2009) suppresses MFS in the inner molecular layer of the dentate gyrus and might also be expected to inhibit MFS in the hilus. It seems likely that synaptic reorganization of DGCs and CA3 pyramidal cells contributes substantially to the change in hilar interneuron excitability. Perhaps surprisingly, removal of CA3 pyramidal neurons failed to alter the pattern of EPSC frequency changes (i.e., enhanced after CCI injury and intermediate in rapamycin-treated mice). However, removal of CA3 would only eliminate synaptic excitation due to action potentials, and action potential-independent vesicle release from presynaptic terminals would be preserved. Thus, the patterns of excitatory synaptic reorganization imply formation of new synapses. This is consistent with previous work demonstrating that most synaptic excitation in acute slices is action potential-independent (Burke, 1967; Brown et al., 1979), and that action potential-independent, miniature EPSC frequency is elevated in eGFP+ hilar neurons ipsilateral to CCI injury (Hunt et al., 2011). Whereas rapamycin treatment normalized input arising after activation of DGCs, consistent with effects of mTOR inhibition on MFS and synaptic reorganization of DGCs, there was no reduction in excitatory synaptic responses arising after stimulation of CA3 pyramidal neurons, which could account for the intermediate excitability level of hilar interneurons in rapamycin-treated mice. These results also suggest that reactive plasticity originating from CA3 pyramidal cells after CCI injury occurs independent of mTOR function.

The present approach with glutamate photolysis cannot distinguish mono-synaptic versus poly-synaptic connections between either activated DGCs or activated CA3 pyramidal cells and their downstream DGC or hilar interneuron targets. Postinjury connectivity of CA3 pyramidal cells is not limited to the somatostatin-positive hilar inhibitory interneurons, and backprojections to DGCs have been proposed as a potential form of hippocampal reorganization in epilepsy (Scharfman, 2007). In this study, glutamate photostimulation of the CA3 pyramidal cell layer revealed relatively sparse synaptic connectivity with DGCs in all experimental groups, even in slices where the hippocampal circuits were disinhibited to expose recurrent connections between pyramidal cells (Wong and Traub, 1983). Recurrent connections between DGCs are increased after CCI and could also contribute to polysynaptic activation of hilar interneurons during CA3 photostimulation in the injured hemisphere. Even in the presence of disinhibited recurrent connections between principal neurons, however, CA3-to-DGC connections were rarely detected, consistent with previous work (Scharfman, 1994a). Hilar mossy cells might also participate in polysynaptic activation of GFP+ inhibitory interneurons of DGCs after glutamate photolysis in the reactive circuits studied here. Mossy cells receive connections from both CA3 pyramidal neurons and DGCs, and they innervate DGCs and hilar interneurons (Scharfman, 1994a,c, 1995; Larimer and Strowbridge, 2008). This seems unlikely because the axonal morphology of mossy cells suggests minimal participation in connections with DGCs within the 350-µm brain slices used here (Buckmaster et al., 1996; Larimer and Strowbridge, 2008). Mossy cells make synaptic contacts with nearby hilar neurons (Buckmaster et al., 1996) and although a large percentage of mossy cells are killed after seizures or moderate brain trauma (Santhakumar et al., 2000; Deng and Xu, 2011), surviving mossy cells elaborate structurally in a model of TLE (Zhang et al., 2015). Mossy cells can extend small portions of their dendritic tree into both CA3 and the granule cell layer (Zhang et al., 2015), so stimulation of either site could potentially activate a mossy cell dendrite directly. That stated, rapamycin effectively reduced responses to photostimulation in the granule cell layer, but not the CA3 pyramidal cell layer. If mossy cell connections with hilar interneurons mediated the responses to photostimulation, then they should have persisted after stimulation of the granule cell layer, which was not the case. Thus, the present interpretation that downstream responses observed during CA3/DG photoactivation occurred by monosynaptic connections is consistent with all of this information; however, it cannot rule out the possibility of additional intermediate cells giving rise to polysynaptic activation.

The relative selectivity of rapamycin’s effect on reorganization of synaptic input from DGCs could be related to the suppression of DGC neurogenesis after CCI in rapamycin treated mice (Butler et al., 2015). Adult born DGCs are hypothesized to contribute substantially to abnormal synaptic connectivity within the dentate gyrus after an epileptogenic insult (Parent et al., 1997), and adult born DGCs strongly innervate inhibitory interneurons in the absence of brain injury (Drew et al., 2016). Rapamycin’s suppression of injury-induced neurogenesis may account for the prevention of DGC contributions to synaptic reorganization, while preserving the rapamycin-resistant increase in input from CA3 pyramidal cells, especially if a large proportion of the synaptic reorganization is due to axons arising from adult born neurons. Thus, although rapamycin might restrain MFS in adult DGCs, a suppressive effect on adult neurogenesis could account for the relatively selective effects on DGC, versus CA3 pyramidal cell, input to hilar interneurons after focal brain injury.

Additional possible mechanisms could account for rapamycin’s effect on synaptic reorganization of input to hilar inhibitory interneurons, including effects on metabolism and cell growth. The pathologic weight gain that develops after SE in mice is reversed with chronic rapamycin treatment (Hester et al., 2016). Here, there was no difference in weight gain between sham-treated and CCI-injured mice, but chronic rapamycin treatment after CCI injury reduced the amount of weight gained in all groups, similar to previous reports of altered growth curves in naïve and disease rodent models (Deblon et al., 2012; Staats et al., 2013; Hebert et al., 2014; Hsieh et al., 2016). In addition, somatic size of somatostatinergic hilar interneurons increased after pilocarpine-induced SE, compared to control mice, and rapamycin treatment suppressed this increase in soma size (Buckmaster and Wen, 2011). CCI injury did not significantly alter whole-cell capacitance, and daily rapamycin treatment reduced both contralateral and ipsilateral whole-cell capacitance of eGFP+ interneurons after injury, consistent with suppressive effects of mTOR inhibition on cellular growth (Heitman et al., 1991). Still, no functional measure from neurons contralateral to injury was significantly different from those in sham-injured controls, regardless of rapamycin treatment, suggesting that effects on metabolism or cell growth were not the primary mediators of rapamycin’s effect on cellular or synaptic excitability of these neurons.

Rapamycin treatment inhibits synaptic reorganization arising from DGCs, but not CA3 pyramids following CCI injury, resulting in an intermediate level of synaptic excitation in hilar inhibitory interneurons. A lack of effect on reorganization of inputs arising from CA3 also suggests different roles for mTOR in mediating reactive plasticity of DGCs and CA3 pyramidal cells, and this is consistent with rapamycin’s suppressive effect on adult neurogenesis after brain injury. Sustained synaptic reorganization of CA3 pyramidal cells, or other aberrant circuits, could contribute to the reemergence of epileptogenesis in models of TLE and PTE when rapamycin treatment is ceased, even in the absence of significant MFS (Guo et al., 2013; Heng et al., 2013). Blocking mTOR signaling modifies, but does not abrogate, the complex reorganization of cortical synaptic circuitry after TBI, highlighting the need to better understand the cellular changes relevant to posttraumatic epileptogenesis and functional recovery.

## Synthesis

Reviewing Editor: Lorna Role, Stony Brook Decisions are customarily a result of the Reviewing Editor and the peer reviewers coming together and discussing their recommendations until a consensus is reached. When revisions are invited, a fact-based synthesis statement explaining their decision and outlining what is needed to prepare a revision will be listed below. The following reviewer(s) agreed to reveal their identity: Vijayalakshmi Santhakumar.

Synthesis for eN-TNWR-0134-17X Brain injury-induced synaptic reorganization in hilar inhibitory neurons is differentially suppressed by rapamycin

This manuscript is significantly improved based on prior reviews from its submission to JNS. As it stands the experiments are technically sound and prior rounds of revisions have fully addressed technical and analytical concerns.

The manuscript is also improved in that it now highlights the central novel result that rapamycin treatment selectively prevents CCI induced increases in afferentation of eGFP+ neurons originating from DCGs, without altering CCI induced increases in afferentation originating from CA3 pyramidal neurons. The implication is that CA3 neurons drive a rapamycin-resistant increase in SOM neuron sEPSCs after brain injury. However, this is not experimentally verified.

We generally agree that (as the authors contend), these data could plausibly help promote development of additional specific and testable hypotheses that could improve our understanding of post injury neuroplasticity and/or of epileptogenisis.

Nevertheless the reviewers believe the impact of the paper in its current form is likely to be relatively low to moderate and could be significantly improved with the addition of one set of experiments to address the underlying mechanism of the differential effects of rapamycin on CCI induced increases in afferentation from the granule cell layer vs. CA3.

The observation that the effect of CA3 stimulation on SOM current responses is resistant to rapamycin is intriguing. Is this due to sprouting/formation of new synapses specifically from CA3 neurons? At a minimum the authors should address whether the continued ability of CA3 stimulation to activate (mono or polysynaptic) SOM current responses after treatment with rapamycin actually contributes to a demonstrable post-injury increase in SOM neuron sEPSCs or firing.

Specifically, we ask that the authors examine if the increase in sEPSC frequency in SOM neurons in slices from rapamycin treated animals requires CA3.

To do this one would examine the sEPSC frequency in sham/CCI/CCI-rapa in coronal or transverse hippocampal slices with CA3 intact (cut between CA1 and CA3- thus replicating data in fig 2) and in a subgroup of slices from the same mice following micro-dissection of CA3.

## Author Response

Responses to reviewer comment.

Reviewer comment: At a minimum the authors should address whether the continued ability of CA3 stimulation to activate (mono or polysynaptic) SOM current responses after treatment with rapamycin actually contributes to a demonstrable post-injury increase in SOM neuron sEPSCs or firing.

Specifically, we ask that the authors examine if the increase in sEPSC frequency in SOM neurons in slices from rapamycin treated animals requires CA3.

To do this one would examine the sEPSC frequency in sham/CCI/CCI-rapa in coronal or transverse hippocampal slices with CA3 intact (cut between CA1 and CA3- thus replicating data in fig 2) and in a subgroup of slices from the same mice following micro-dissection of CA3.

Authors’ response 1: We performed the experiment suggested by the reviewer, in which recordings were made from GFP+ hilar interneurons from slices after microdissection of CA3. Slices were made from sham or CCI-injured mice and subsequently treated with either vehicle or rapamycin for 8-12 weeks, exactly as in the initial version of the manuscript. Results indicated that a similar pattern of sEPSC frequency changes arose as in slices in which CA3 was intact. Details of results arising from these experiments are presented in the manuscript text and discussed in the context of previous findings and the other results in the paper. Essentially, removing CA3 did very little to the patterns of group-related changes. However, this should not be too surprising, since action potential-independent terminal release (in the form of mEPSCs) to hilar interneurons is increased after CCI and SE, suggesting the addition of new synapses (Halabisky et al., 2010; Hunt et al., 2011). Dissecting CA3 should not necessarily be predicted to obviate the changes in sEPSC frequency (except for a relatively few action potential-mediated events) because the synaptic terminals cannot be removed (i.e., EPSCs measured after CA3 isolation would include those due to synaptic release from new terminal contacts of (cut) CA3 axons). Thus, although perhaps less satisfying than the reviewer had hoped, the new data add value to the paper. The preservation of EPSCs evoked after stimulation of CA3 pyramidal cell bodies after rapamycin treatment (i.e., the primary result) is consistent with the continued increase in excitatory synaptic input to these cells. Thus, photoactivation of CA3 pyramidal cells is sufficient to drive excitatory synaptic input to hilar inhibitory interneurons after brain injury, and this input is unaffected by rapamycin treatment. This does not imply, however, that the increase in excitatory drive is exclusively or directly from CA3 pyramidal cells, since projections to SOM interneurons could also arise from other cells that are driven by increased CA3 pyramidal cell activity, and this is discussed in the manuscript.

Authors’ response 2: An additional experiment was performed, which was suggested in an earlier review. Namely, the effect of ionotropic glutamate receptor blockade on action potential firing in GFP+ hilar interneurons was examined. Blocking glutamate receptors significantly reduced firing rate. This result and appropriate discussion is now included in the manuscript.
